# Therapeutic potential of formononetin in cirrhotic portal hypertension: modulating hepatic fibrosis, macrophage polarization, and lymphangiogenesis

**DOI:** 10.3389/fimmu.2025.1571007

**Published:** 2025-08-07

**Authors:** Zhenghao Wu, Guqing Luo, Hongjie Li, Min Chen, Guangbo Wu, Qiang Fan, Chihao Zhang, Jiwei Yu, Jiayun Lin, Jinbo Zhao, Xiaoliang Qi, Haizhong Huo, Meng Luo, Lei Zheng

**Affiliations:** Department of General Surgery, Shanghai Ninth People’s Hospital, Shanghai Jiaotong University School of Medicine, Shanghai, China

**Keywords:** portal hypertension, liver cirrhosis, macrophages polarization, lymphangiogenesis, formononetin

## Abstract

**Introduction:**

Formononetin regulates intrahepatic fibrogenesis and macrophage polarization to improve portal pressure and liver function in rats with cirrhotic PHT through SMAD3, STAT1, STAT3 and GSK-3β signaling pathways, and modulates lymphangiogenesis through direct action and macrophage polarization-mediated indirect effects. Formononetin has the potential function of treating cirrhotic PHT.

**Background and aims:**

Formononetin (FN) has been reported to have anti-fibrotic effects in the kidneys and anti-M1 polarization effects on macrophages. Lymphangiogenesis is closely associated with macrophages, but its role in cirrhosis and portal hypertension (PHT) remains unclear. This study aims to explore the effects of FN on cirrhotic PHT and the disease-related intrahepatic lymphangiogenesis.

**Methods:**

*In vivo* experiments, bile duct ligation (BDL) induced PHT models were conducted in rats, followed by a 4-week FN treatment (50 mg/kg/day) administered via gavage. Hemodynamics, liver fibrosis, infiltration and polarization of intrahepatic macrophages, as well as intrahepatic lymphangiogenesis, were observed. For *in vitro* experiments, hepatic stellate cells, macrophages and lymphatic endothelial cells were used to observe the multiple effects of FN.

**Results:**

FN significantly ameliorated portal pressure in cirrhosis-induced PHT, improved liver function, and reduced liver fibrosis and hepatic stellate cell activation with decreased SMAD3 protein expression. The intrahepatic macrophage infiltration were markedly decreased, and M1-type macrophage polarization was suppressed by FN, accompanied by inhibition of STAT1, STAT3 and GSK-3β signaling pathways. In PHT models, FN reduced VEGF-C and VEGF-D levels in both the liver and blood, inhibiting intrahepatic lymphangiogenesis in portal area. *In vitro*, high-dose FN significantly inhibited lymphatic endothelial cell proliferation, while the pro-lymphangiogenic effect of conditioned medium from FN-treated M1 macrophages was diminished.

**Conclusions:**

FN significantly ameliorates cirrhotic PHT while reducing fibrosis, suppressing macrophage M1 polarization and inhibiting lymphangiogenesis. This may result from its modulation of multiple signaling pathways (SMAD3, STAT1, STAT3 and GSK-3β).

## Graphical Abstract

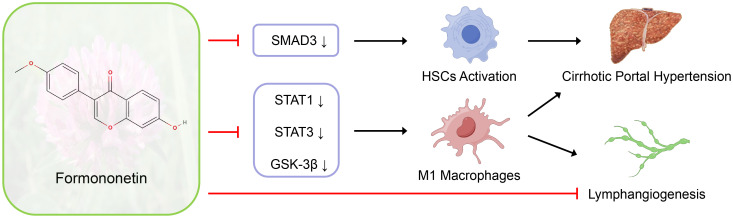

## Introduction

Portal hypertension (PHT), a defining complication of decompensated cirrhosis, manifests clinically through ascites, splenomegaly, and the development of portosystemic collateral circulation. The pathogenesis of PHT is principally driven by two interrelated mechanisms, cytokine-mediated hepatic stellate cell (HSC) activation and liver sinusoidal endothelial cell (LSEC) dysfunction, both hallmarks of cirrhotic progression (1). HSC activation precipitates excessive extracellular matrix deposition, whereas LSEC dysfunction predominantly elevates intrahepatic vascular tone. In the cirrhotic microenvironment, both resident Kupffer cells and recruited bone marrow-derived macrophages undergo dynamic polarization, predominantly adopting pro-inflammatory M1 or anti-inflammatory M2 phenotypes that differentially regulate disease progression (2). Progressive cirrhosis exacerbates portal pressure (PP) through multifactorial mechanisms: disrupted nitric oxide homeostasis, mesenteric arterial vasodilation and remodeling, extensive collateralization, and ascites accumulation.

Despite contributing 25-50% of thoracic duct lymph flow (3), the hepatic lymphatic system remains understudied, primarily due to historical limitations in definitive lymphatic endothelial markers. Lymphangiogenesis, the formation of new lymphatic vessels from pre-existing ones, is widely observed in various liver pathologies and is predominantly driven by the VEGF-C/VEGFR3 signaling pathway (3). While conventionally viewed as adaptive through its roles in interstitial drainage, immune surveillance, and inflammatory resolution (4, 5), the functional consequences of lymphangiogenesis in PHT remain mechanistically unresolved.

**Figure 1:**
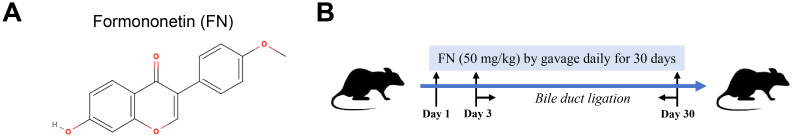
*In vivo* study design for evaluating FN effects. **(A)** The molecular formula of FN. **(B)** Construction of rat model of PHT and FN administration.

Liver transplantation, while definitive for end-stage PHT, faces critical constraints including donor organ scarcity, disease progression kinetics, and socioeconomic disparities in access (6, 7). This therapeutic gap underscores the imperative for pharmacological interventions. Drug repurposing strategies offer particular translational advantages given their established safety profiles and accelerated development timelines. Formononetin (FN), a bioactive isoflavone (**Figure 1A**), exhibits pleiotropic properties including estrogen receptor modulation, antifibrotic activity, and antioxidant effects (8, 9), positioning it as a compelling candidate for PHT management. However, its therapeutic potential and mechanistic underpinnings remain unexplored.

In this study, we will explore the potential therapeutic effects of FN on PHT by alleviating liver fibrosis, inflammation, macrophage infiltration and M1 polarization, as well as its mechanism of influencing intrahepatic lymphangiogenesis.

## Methods

### Animals experiments

Male Sprague Dawley (SD) rats, aged 6–8 weeks and weighing 250–300 g, were obtained from the Experimental Animal Center of the School of Medicine, Shanghai Jiao Tong University (Shanghai, China). The rats were housed in specific pathogen-free facility under controlled conditions (22°C, 40%–60% humidity and 12-h light/dark cycle) with free access to food and water. Ethical approval for all animal-related protocols was granted by the Ethics Committee of Shanghai Ninth People’s Hospital, Shanghai Jiao Tong University School of Medicine (approval no. SH9H-2019-A201-1).

The rats underwent either a sham operation (Sham) or common bile duct ligation (BDL) as previously described (10), and were randomly assigned to three groups: (a) Sham group (n = 8), (b) BDL group (n = 12), (c) BDL + FN (50 mg/kg/day) group (n = 16). FN powder (#F141481, Aladdin, China) was dissolved in normal saline containing 0.5% carboxymethylcellulose sodium salt (CMC-Na, #C304951, Aladdin, China) to form a stable milky solution. Over a 30-day period, rats in the experimental group received daily FN administration via oral gavage, followed by BDL surgery on day 3, as shown in **Figure 1B**. Control group rats received daily vehicle gavage and sham surgery.

At the experimental endpoint, rats were weighed and anesthetized via intraperitoneal injection of tribromoethanol (300 mg/kg, 2.5% solution). Briefly, we punctured the PE50 tube into the left femoral artery or portal vein of the rats. After connecting the tube to the pressure sensor, the mean arterial pressure (MAP) and portal vein pressure were measured using an ALC-MPA multi-channel biological information analysis system (Shanghai Alcott Biotech Co., Ltd., Shanghai, China). Once the measurements were complete, rats were euthanized by exsanguination under maintained anesthesia. Then liver tissues and serum samples were collected. The serum samples were subsequently analyzed for the levels of alanine transaminase (ALT), aspartate Aminotransferase (AST) and total bilirubin (TBIL) using an automatic clinical analyzer (HITACHI Ltd, Japan).

### Histological and immunohistochemical analysis

Rat hepatic lobes were obtained and fixed in a 10% formalin buffer solution with a pH of 7.4, followed by embedding in paraffin blocks for histological analysis. Subsequently, the liver sections underwent staining using the hematoxylin and eosin (H&E), Masson’s trichrome, and Sirius red techniques.

IHC examination was conducted on the rehydrated paraffin-embedded live sections. Endogenous peroxidase was inactivated in methanol solution containing 0.3% H_2_O_2_. Citrate buffer was used for antigen retrieval. Incubation with 10% goat serum (#G1208, Servicebio, China) for 1 h at 37°C was performed to prevent nonspecific antibody binding. The sections were then incubated overnight at 4°C with anti-CD68 antibody (1:200, #ab283654, Abcam, UK). Sections were then washed, followed by incubation with secondary antibodies (1:1000, #31460, Invitrogen, USA) for 1 h at room temperature. The bound antibodies were visualized using diaminobenzidine (DAB) as the chromogen, followed by restaining with hematoxylin.

### Immunofluorescence staining

For IF analysis, rehydrated paraffin-embedded live sections or fixed cells were treated with 0.3% Trition X-100 (#T8787, Sigma-Aldrich, USA) for 15 min and then blocked in 10% goat serum (#G1208, Servicebio, China) for 1 h at 37°C. The sections were then incubated overnight at 4°C with anti-CD68 antibody (1:50, #ab955, Abcam, UK) plus anti-iNOS antibody (1:50, #ab283655, Abcam, UK) or anti-CD163 antibody (1:200, #ab182422, Abcam, UK), anti-α-SMA antibody (1:100, #ab7817, Abcam, UK) plus anti-LYVE-1 antibody (1:100, #AF7939, R&D Systems, USA), or anti-iNOS antibody (1:100, #CY5993, Abways, China) plus anti-CD206 antibody (1:100, #60143-1-Ig, Proteintech, USA). Samples were then washed, and secondary fluorescent antibodies (1:1000, #A-21235, #A-11008, #A-21448, #A-21245, #A-11001, Invitrogen, USA) were prepared for incubation at room temperature in dark for 1 h. After washing, antifade mounting medium with DAPI (#G1407 Servicebio, China) was added to the samples for nuclei staining. Images were captured under a fluorescence microscopy.

### Determination of lymphatic vessel density and lymphatic vessel area

Based on double IF staining of α-SMA and LYVE-1, the portal areas and portal veins (PV) were identified by scanning liver tissues at low magnification (x40). The lymphatic vessels (LYVE-1^+^α-SMA^-^) around PV (α-SMA^+^) exhibited distinct differences in structure and position compared to the nearby hepatic sinusoids. The numbers and covered area of lymphatic vessels were measured using ImageJ software in a single x400 field, and four portal areas were randomly selected per slice. The results were adjusted for the area of the relevant PV as percentage for LVD and LVA.

### Enzyme-linked immunosorbent assay

Rat serum and supernatants from liver tissue homogenates were collected for cytokine detection. The levels of secreted VEGF-C and VEGF-D were measured using rat-specific ELISA kits obtained from Jiangsu Meimian Industrial Co., Ltd., following the manufacturer’s protocols. Absorbance at 450 nm was read using a microplate reader (BioTek, USA), and cytokine concentrations were calculated based on the standard curves.

### Cell culture

Rat HSC-T6 (hepatic stellate cells) and mouse Raw264.7 (macrophages) cells were procured from Pricella LifeScience & Technology Co., Ltd. and cultured in DMEM (#SH30022.01, Cytiva, USA) with 10% FBS (#SH30070.03, Cytiva, USA). HSC-T6 cells were seeded in 6-well plates at 3x10^5^ cells per well overnight. The cells were starved for 4 h without FBS, followed by incubation with TGF-β1 (20 ng/ml, #100-21C, PeproTech, USA) or FN (80 or 160 μM) for an additional 48 h before collection.

Raw264.7 cells were seeded in 6-well plates at 3x10^5^ cells per well overnight. The cells were incubated with LPS (50 ng/ml, #L2880, Sigma-Aldrich, USA) plus IFN-γ (40 ng/ml, #315-05, PeproTech, USA), or FN (80 or 160 μM) for another 24 h before collection. For conditioned medium (CM), Raw264.7 cells were cultured until reaching over 60% confluency and stimulated as indicated for 24 h. The cells were meticulously washed multiple times with PBS, and then incubated with 0.5% FBS DMEM for another 24 h. The CM was collected, centrifuged to remove cell debris, and stored at -20°C.

Human lymphatic endothelial cells (hLECs) immortalized by SV40 were obtained from Cellverse Bioscience Technology Co., Ltd. and cultured in endothelial cell medium (ECM, #1001, ScienCell, USA). hLECs were seeded in 6-well plates at 1x10^5^ cells per well overnight, and exposed to FN (20, 40 or 80 μM) for another 24 h. To investigate the paracrine effects of macrophages, hLEC were incubated with rVEGF-C (50 ng/ml, #589704, BioLegend, USA) or ECM plus equal volumes of CM (from M0, M1, or FN-treated M1 macrophages) for 24 h after overnight culture.

### CCK-8 assay

The viability of HSC, macrophage and hLEC was assessed using CCK-8 assay (#C0037, Beyotime, China). Cells were seeded in 96-well plates at 5x10^3^ cells/well (4 replicates) and exposed to FN at increasing concentrations (0, 20, 40, 80, 160 μM) for varying durations (0, 24, 48 and 72 h). Then the original culture mediums were replaced with 100μL of basal medium containing 10 μL of CCK-8 reagent and cultivated for 1 h at 37 in the dark. Microplate reader (BioTek, USA) was used to detect the absorbance of mixtures at 450 nm. The experimental conditions were adapted from established FN protocols (11).

### Western blot analysis

Liver tissues and cells were lysed in RIPA buffer (#P0013C, Beyotime, China) containing protease inhibitors (#ST2573, Beyotime, China) and phosphatase inhibitors (#G2007, Servicebio, China). The protein samples were separated by sodium dodecyl sulfate polyacrylamide gel (7.5-10%, #PG211 and #PG212, Epizyme Biotech, China) electrophoresis (SDS-PAGE) and transferred to polyvinylidene fluoride (PVDF) membranes (0.45 μm, #PR05509, Millipore, USA), followed by blocking with 10% skim milk and incubation with antibodies against GAPDH (1:10000, #60004-1-Ig, ProteinTech, USA), α-SMA (1:2000, #GB11044, Servicebio, China), COL1A1 (1:1000, #GB11022, Servicebio, China), TGF-β1 (1:2000, #A2124, ABclonal, China), SMAD2 (1:2000, #ab40855, Abcam, UK), p-SMAD2 (Ser255) (1:2000, #CY5859, Abways, China), SMAD3 (1:2000, #ab28379, Abcam, UK), p-SMAD3 (Ser423 and Ser425) (1:2000, #ab52903, Abcam, UK), IL-1R (1:1000, #ab106278, Abcam, UK), IL-1Ra (1:10000, #ab124962, Abcam, UK), GSK3β (1:2000, #CY2434, Abways, China), p-GSK3β (Ser9) (1:2000, #CY6248, Abways, China), STAT1 (1:2000, #CY5227, Abways, China), p-STAT1 (Tyr701) (1:2000, #CY5917, Abways, China), STAT3 (1:2000, #CY5165, Abways, China) and p-STAT3 (Tyr705) (1:2000, #CY6566, Abways, China), at 4°C overnight. Following multiple washes with TBST, and PVDF membranes were incubated with secondary antibody (1:50000, #SB-AB0101 and #SB-AB0102, ShareBio, China) at room temperature for 1 h, and detected with ECL substrate (#SB-WB004, ShareBio, China) using Chemi-Image System (#5200CE, Tanon, China). ImageJ software was used to analyze the intensity of protein bands.

### Real-time quantitative PCR analysis

Total RNA was extracted from liver tissues and cells using Trizol reagent (#R401-01, Vazyme, China). Complementary DNA was synthesized by the reverse transcription kit (#R323-01, Vazyme, China). Real-time PCR was performed using qPCR Master Mix (#B21203, Selleck, USA) on the LightCycler^®^ 480 II Real-time PCR System (Roche, Germany). Primers used in RT-PCR was listed in **Supplementary Table S1** and synthesized by Sangon Biotech (Shanghai) Co., Ltd. The house-keeping gene (*GAPDH*) was employed for expression normalization. The relative expression of detected genes was calculated using the 2^-ΔΔCt^ method.

### Flow cytometry

Treated macrophages were digested into single cell suspensions using accutase (#07920, StemCell, USA) and diluted to 1x10^6^ cells/ml in FACS buffer. The following sequential staining procedures were conducted: viability staining (FVS780, #565388, BD Biosciences, USA), Fc receptor blocking (Anti-Mouse CD16/CD32, #553141, BD Biosciences, USA), surface marker staining (PE Anti-Mouse CD86, #553692, BD Biosciences, USA), fixation/permeabilization, and nuclear marker staining (AF647 Anti-Mouse CD206, # 565250, BD Biosciences, USA). The data were acquired on a BD LSRFortessa X-20 (BD Biosciences, USA) and analyzed with FlowJo v10.0.7 software. The gating strategy is presented in the **Supplementary Materials** (**Supplementary Figure S1**).

### Tube formation assay

hLECs were cultured in ECM until reaching over 80% confluency, following which the culture medium was changed to DMEM with 0.5% FBS for serum starvation overnight. Subsequently, cells were reseeded in 96-well plates coated with 50μl Matrigel (#0827015, Mogengel Bio, China) at 3x10^4^ cells per well. For testing the direct effect of FN, cells were divided into four groups: (a) DMEM (0.5% FBS and 0.1% DMSO), (b) DMEM (0.5% FBS and 0.1% DMSO) plus rVEGF-C (50 ng/ml), (c, d) DMEM (0.5% FBS and 0.1%DMSO) containing 40 or 80 μM FN. For evaluating indirect effects of FN, cells were assigned to five groups: (a) DMEM (0.5% FBS), (b) DMEM (0.5% FBS) plus rVEGF-C (50 ng/ml), (c) DMEM (0.5% FBS) plus equal volumes of CM from M0 macrophages, (d) DMEM (0.5% FBS) plus equal volumes of CM from M1 macrophages, (e) DMEM (0.5% FBS) plus equal volumes of CM from FN-treated M1 macrophages. The cells were incubated at 37 °C for 4 h and microscopically analyzed in 5 different fields per well. Tube formation was assessed by the average total length of tubes using ImageJ software with the angiogenesis analyzer plugin.

### Statistical analysis

The results were presented as mean ± S.E.M. Statistical significance was determined using One-way ANOVA with Tukey’s Test. GraphPad Prism 9 was employed for data visualization. A p-value < 0.05 was considered statistically significant for all analyses. All authors had access to the study data and had reviewed and approved the final manuscript.

## Results

### FN alleviated portal pressure and improve liver function in BDL-induced PHT model

**Figure 2:**
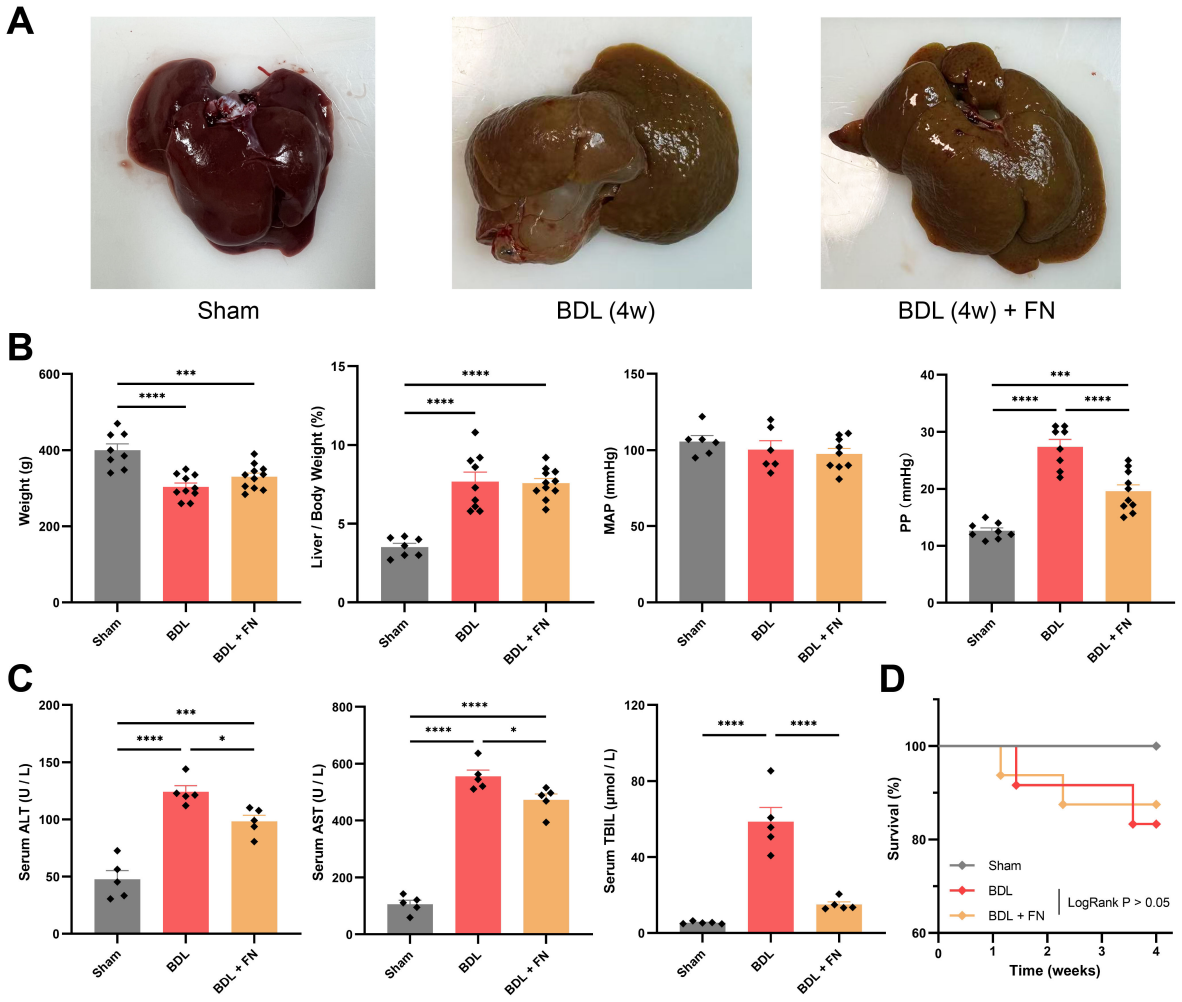
Effects of FN on PHT and liver function in BDL-induced PHT rats. **(A)** Gross liver images of rats from three experimental groups. **(B)** General conditions and hemodynamic status of rats in each group. **(C)** Levels of blood serum ALT, serum AST and serum TBIL of rats in each group. **(D)** Survival curve for each group after surgery 28 days. For Sham group, n = 8, for BDL group, n = 12, for BDL + FN group, n = 16. Data expressed as mean ± S.E.M. Each diamond represents one biological replicate. *p < 0.05; **p < 0.01; ***p < 0.001; ****p < 0.0001 (One-way ANOVA with Tukey’s Test).

On the 28th day post-surgery, the experimental rats were sacrificed, hemodynamic parameters were measured, and liver and serum samples were collected. BDL Rats exhibited significant hepatomegaly with cholestatic pigmentation, diffuse nodular projections on the hepatic surface, and dilated extrahepatic bile ducts beneath (**Figure 2A**). Compared to the Sham group, the BDL group exhibited a noticeable deterioration in body weight, hepatosomatic index, and portal pressure (**Figure 2B**). In contrast, the FN treatment can improve portal pressure without reduction in systemic blood pressure (**Figure 2B**). Serum ALT, AST and TBIL were dramatically increased in the BDL group compared with the Sham group, which were significantly decreased after the treatment with FN (**Figure 2C**). However, there was no significant difference in survival rates (83.3% for BDL group vs. 87.5% for BDL + FN group) between the FN group and the BDL group (**Figure 2D**).

### FN alleviated liver fibrosis in BDL-induced PHT model and affected multiple signaling pathways

**Figure 3:**
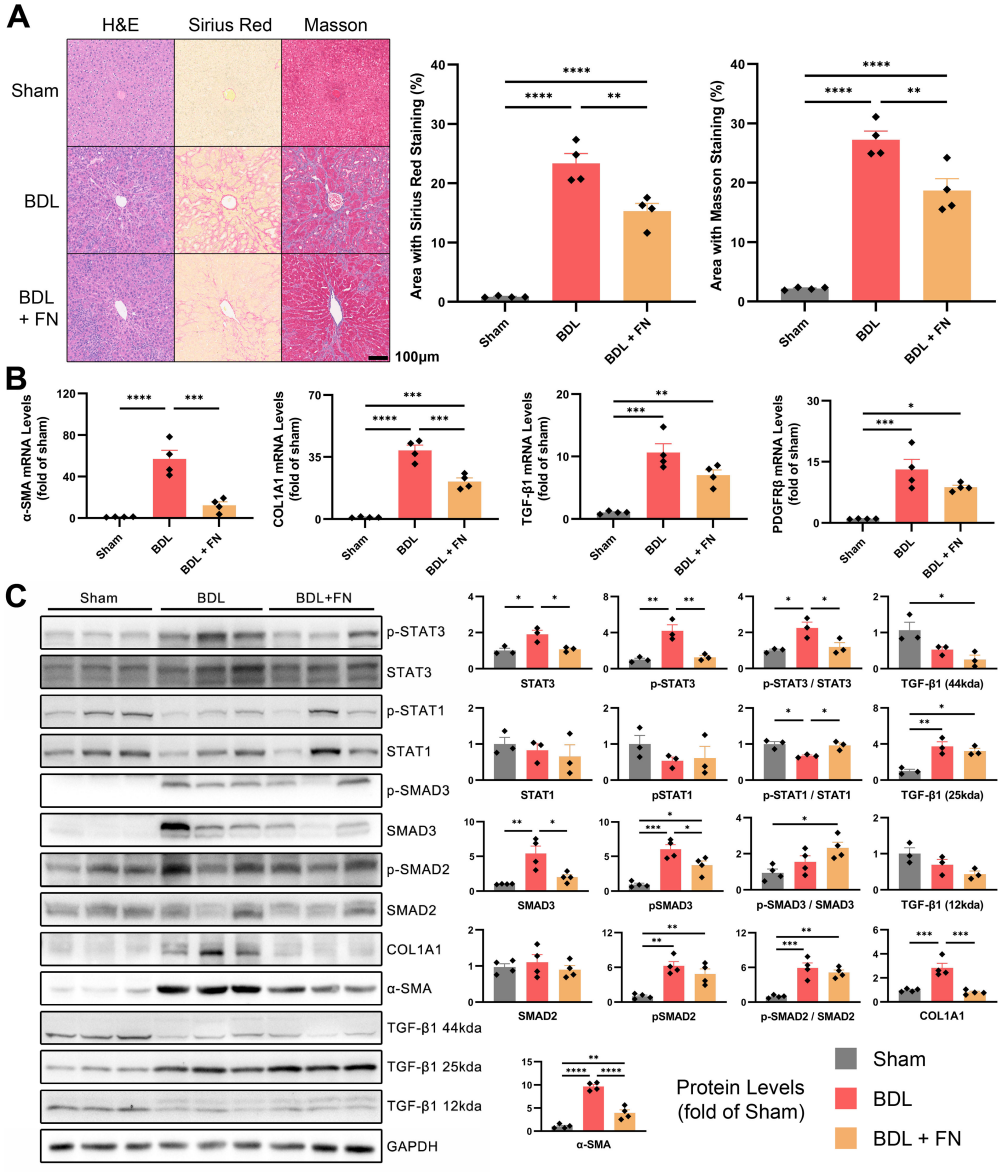
Effects of FN on liver fibrosis in BDL-induced PHT model. **(A)** Histological staining of liver tissue sections using H&E, Sirius red and Masson’s trichrome, and quantitative analysis of the fibrotic area in each groups. **(B)** The mRNA expression of *α-SMA*, *COL1A1*, *TGF-β1* and *PDGFRβ* in liver tissues of each groups. **(C)** Immunoblotting of p-STAT3 (Tyr705), STAT3, p-STAT1 (Tyr701), STAT1, p-SMAD3 (Ser423 and Ser425), SMAD3, p-SMAD2 (Ser255), SMAD2, COL1A1, α-SMA and TGF-β1 (three forms) in liver tissues of each groups. Data expressed as mean ± S.E.M. Each diamond represents one biological replicate. *p < 0.05; **p < 0.01; ***p < 0.001; ****p < 0.0001 (One-way ANOVA with Tukey’s Test).

The degree of liver fibrosis was initially assessed by fibrosis area ratio of liver tissues sections, stained by H&E, Masson’s trichrome and Sirius red (**Figure 3A**). According to the quantitative analysis, FN treatment substantially mitigated liver fibrosis resulting from BDL surgery. Furthermore, this trend was confirmed by the mRNA and protein levels of α-SMA and COL1A1 in liver tissues (**Figures 3B, C**). TGF-β1/Smad signaling, one of the major pro-fibrotic pathways was also evaluated. The mRNA expression of *TGF-β1* and the protein level of active TGF-β1 homodimers (25kda) in the livers of PHT rats were significantly increased, while the protein levels of inactive TGF-β1 monodimers (12kda) and precursor (44kda) were significantly decreased (**Figures 3B, C**). The mRNA of *PDGFRβ*, another key molecule related to fibrosis, were also significantly elevated by BDL treatment (**Figure 3B**). While FN treatment showed no significant effect on TGF-β1 and PDGFRβ, it markedly reduced the elevated protein levels of p-SMAD3 and total SMAD3 (but not SMAD2) observed in the BDL group (**Figure 3C**). Besides that, FN induced a modest but statistically significant elevation in STAT1 phosphorylation (**Figure 3C**), a negative regulator in hepatic fibrosis. In the BDL model, STAT3 was significantly upregulated and underwent phosphorylation-mediated activation, which was suppressed by FN treatment (**Figure 3C**). However, the precise role of STAT3 in liver fibrosis remains debated (12).

### FN alleviated intrahepatic macrophage infiltration and M1 polarization in BDL-induced PHT model

**Figure 4:**
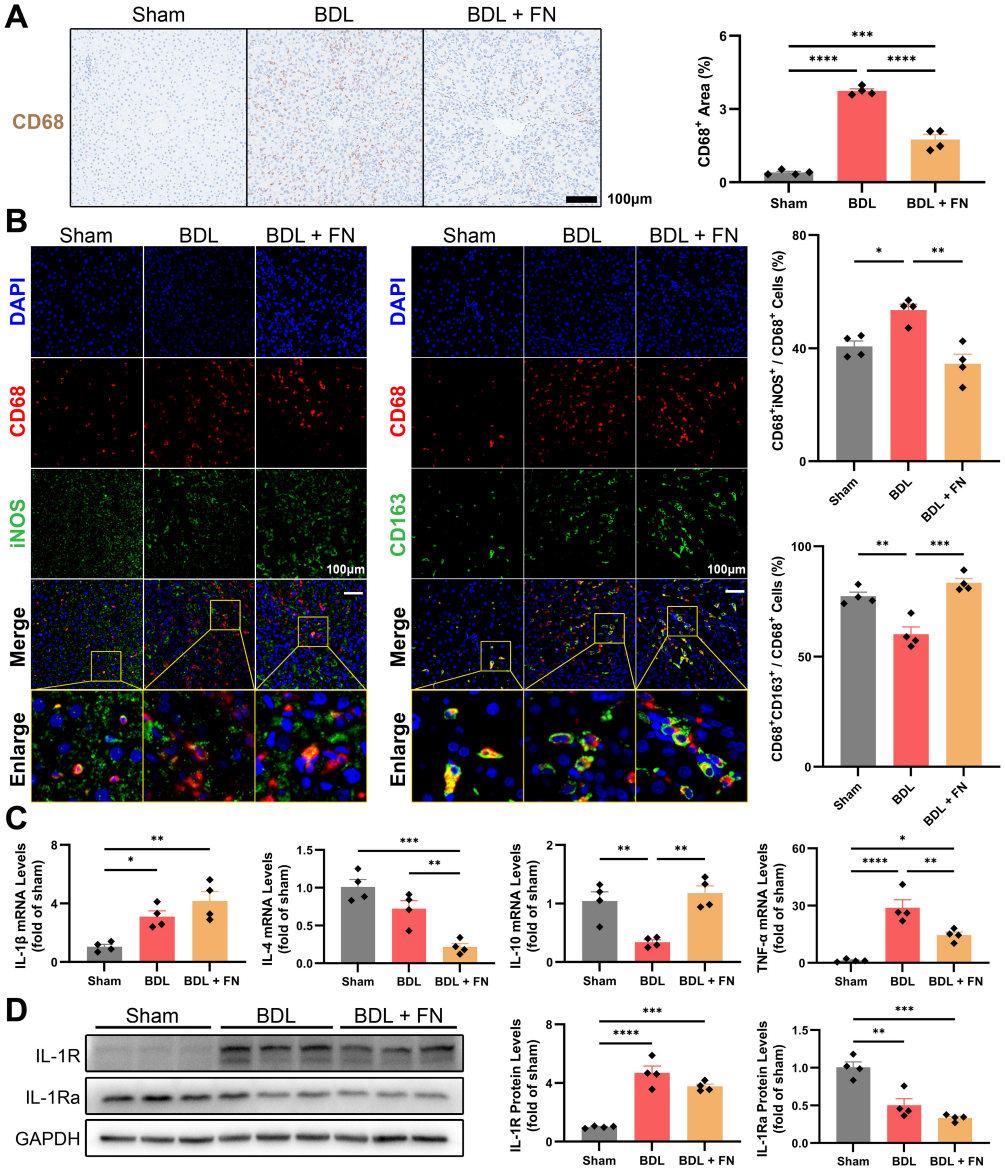
Effects of FN on the intrahepatic infiltration and polarization of macrophages in BDL-induced PHT model. **(A)** Representative immunohistochemistry images of CD68 in liver tissues of each groups, and quantitative analysis of macrophage infiltration. **(B)** Representative immunofluorescence double-staining images of CD68 and iNOS or CD163 in liver tissues of each groups, and quantitative analysis of macrophages polarization (CD68^+^iNOS^+^ for M1-type and CD68^+^CD163^+^ for M2-type). **(C)** The mRNA expression of *IL-1β*, *IL-4*, *IL-10* and *TNF-α* in liver tissues of each groups. **(D)** Immunoblotting of IL-1R and IL-1Ra in liver tissues of each groups. Data expressed as mean ± S.E.M. Each diamond represents one biological replicate. *p < 0.05; **p < 0.01; ***p < 0.001; ****p < 0.0001 (One-way ANOVA with Tukey’s Test).

Macrophage infiltration and polarization phenotype are closely associated with liver fibrosis and lymphangiogenesis. Therefore, we investigated the impact of FN treatment on intrahepatic macrophages in the PHT model. As shown in **Figure 4A**, FN notedly reduced the macrophage infiltration (CD68 positive area) in liver tissue. Furthermore, FN treatment significantly reversed the increase in CD68^+^iNOS^+^ (M1-type) and decrease in CD68^+^CD163^+^ (M2-type) macrophages caused by BDL surgery (**Figure 4B**). FN treatment dramatically improved the mRNA level of *TNF-α*, but showed no improvement in the mRNA level of *IL-1β* (**Figure 4C**). The mRNA and protein levels of IL-1R and IL-1Ra confirmed that FN also had no significant impact on them (**Figure 4D**, **Supplementary Figure S2**). Both IL-4 and IL-10 contribute to M2 phenotype polarization, but FN seemed to exhibit an unexpected preference for *IL-10* and inhibited the expression of *IL-4* (**Figure 4C**).

### FN inhibits intrahepatic lymphangiogenesis in BDL-induced PHT model

**Figure 5:**
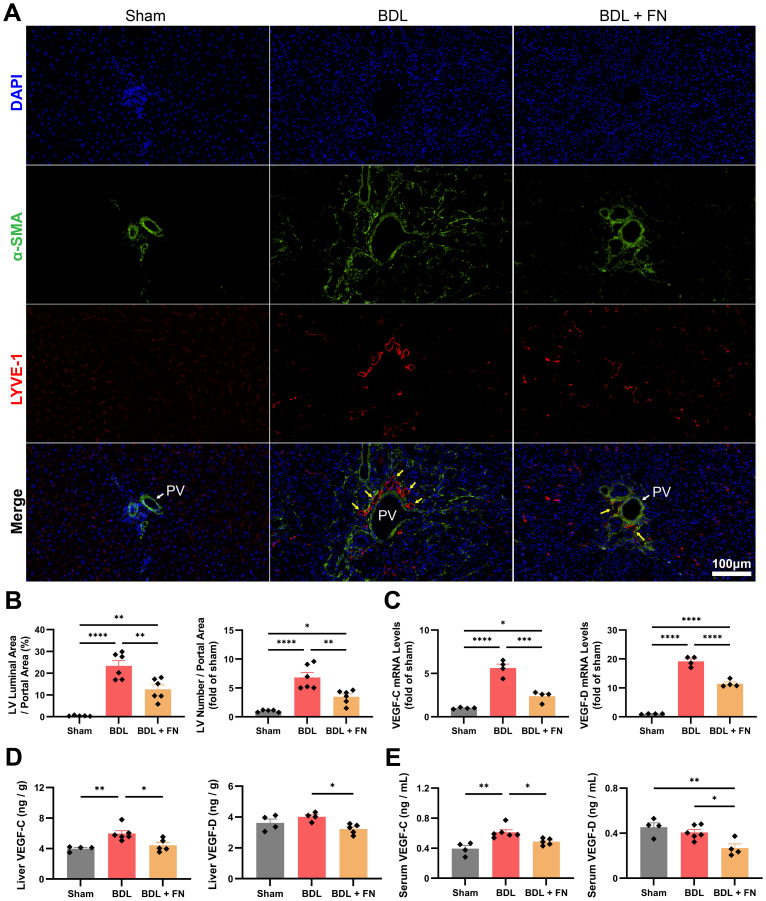
Effects of FN on intrahepatic lymphangiogenesis in BDL-induced PHT model. **(A, B)** Representative immunofluorescence double-staining images of α-SMA and LYVE-1 in portal area, and quantitative analysis of lymphangiogenesis (LYVE-1^+^α-SMA^-^) corrected for portal vein (LYVE-1^-^α-SMA^+^) area. **(C)** The mRNA expression of *VEGF-C* and *VEGF-D* in liver tissues of each groups. **(D, E)** The protein levels of VEGF-C and VEGF-D in the liver and serum determined by ELISA. Data expressed as mean ± S.E.M. Each diamond represents one biological replicate. *p < 0.05; **p < 0.01; ***p < 0.001; ****p < 0.0001 (One-way ANOVA with Tukey’s Test).

Using immunofluorescence double-staining, we observed a significant increase in lymphatic vessels proliferation (LYVE-1^+^α-SMA^-^) around the portal vein (LYVE-1^-^α-SMA^+^) in the portal area following BDL surgery, while FN treatment enormously reduced both the number and area of lymphatic vessels in this region (**Figures 5A, B**). The primary drivers of lymphangiogenesis, VEGF-C and VEGF-D were measured at the mRNA and protein levels in the liver and blood. Compared to the sham group, BDL surgery showed an upsurge in the mRNA and protein levels of VEGF-C and VEGF-D in the liver and blood, while FN treatment markedly suppressed both intrahepatic and extrahepatic levels of VEGF-C and VEGF-D (**Figures 5C–E**).

### FN suppressed TGF-β1-induced activation of HSCs *in vitro*

**Figure 6:**
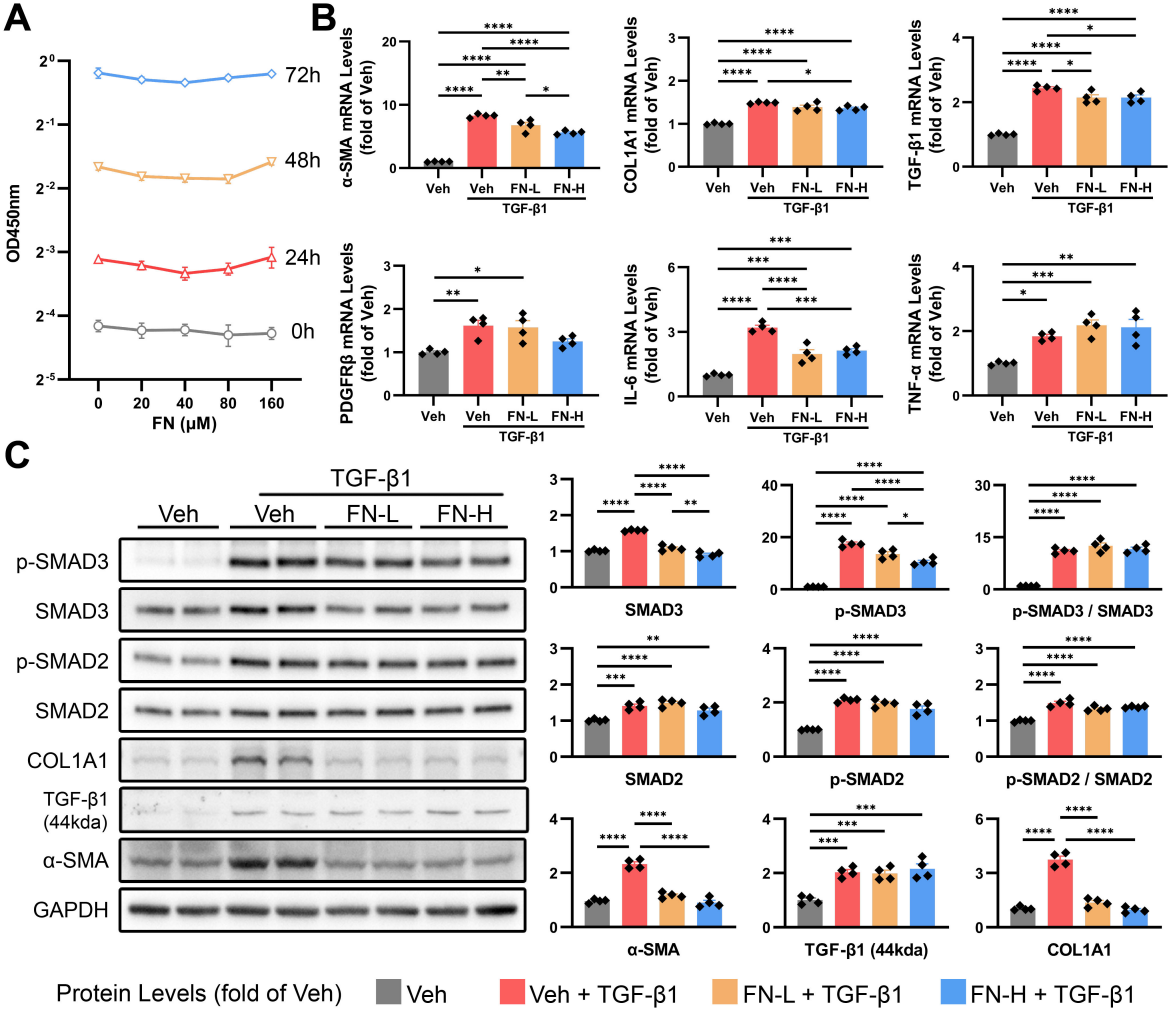
Effects of FN on the activation of HSCs *in vitro*. FN concentration gradients were employed to evaluate its inhibitory effects on HSC activation (induced by 20 ng/ml TGF-β1 for 48 h). FN-L, 80 μM; FN-H, 160 μM. **(A)** CCK-8 assay of HSCs treated with FN (0-160 μM, 0–72 h). **(B)** The mRNA expression of *α-SMA*, *COL1A1*, *TGF-β1*, *PDGFRβ*, *IL-6* and *TNF-α* in treated HSCs of each groups. **(C)** Immunoblotting of p-SMAD3 (Ser423 and Ser425), SMAD3, p-SMAD2 (Ser255), SMAD2, COL1A1, TGF-β1 (44kda) and α-SMA in treated HSCs of each groups. Data expressed as mean ± S.E.M. Each diamond represents one biological replicate. *p < 0.05; **p < 0.01; ***p < 0.001; ****p < 0.0001 (One-way ANOVA with Tukey’s Test).

The activation and proliferation of HSCs induced by liver injury are crucial steps for liver fibrosis. We first evaluated the effect of FN on HSC proliferation and found that FN exhibited no significant inhibitory effect even at high concentrations (**Figure 6A**). The activation of HSCs was induced *in vitro* by TGF-β1 (20 ng/ml), followed by the treatment with FN (80 or 160μM) for 48 h. As shown in **Figure 6B**, FN treatment significantly reduced the mRNA levels of genes associated with HSC activation, including *α-SMA*, *COL1A1*, *TGF-β1*, *PDGFRβ* and *IL-6*, but showed little impact on the expression of *TNF-α*. Regrettably, FN treatment failed to significantly modulate the protein levels of TGF-β1 precursor, consistent with observations in animal models (**Figure 6C**). The canonical TGF-β1 signaling pathway initiates through phosphorylation of receptor-regulated SMAD, especially SMAD2 and SMAD3. *In vitro*, TGF-β1 treatment drastically upregulated the proteins levels of SMAD2 and SMAD3, as well as inducing their phosphorylation (**Figure 6C**). Consistent with *in vivo* findings, FN dose-dependently suppressed p-SMAD3 and total SMAD3, but not SMAD2 (**Figure 6C**).

### FN inhibits M1 polarization of macrophages and reduces the expression of VEGF-C *in vitro*

**Figure 7:**
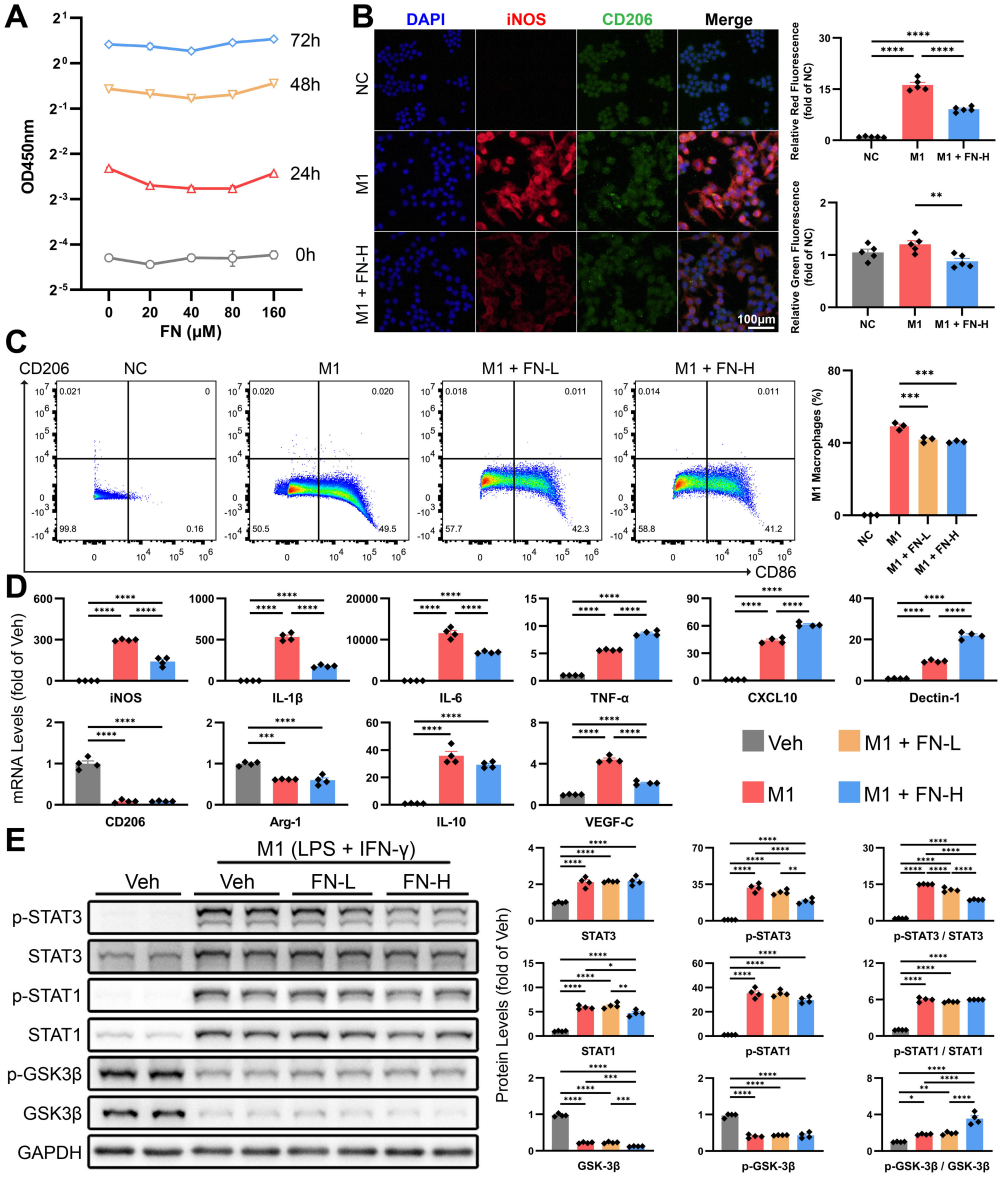
Effects of FN on the polarization of macrophages *in vitro*. Macrophages were exposed to varying concentrations of FN to assess its inhibitory effects on M1 polarization (induced by 50 ng/ml LPS and 40 ng/ml IFN-γ for 24 h). FN-L, 80 μM; FN-H, 160 μM. **(A)** CCK-8 assay of macrophages treated with FN (0-160 μM, 0–72 h). **(B)** Representative immunofluorescence double-staining images of iNOS and CD206, and quantitative analysis of the tendency of macrophage polarization (iNOS for M1-type and CD206 for M2-type). **(C)** Flow cytometry dot plots depict CD86 vs. CD206 expression in FN-treated macrophages. **(D)** The mRNA expression of macrophage polarization markers and *VEGF-C* in treated macrophages of each groups. **(E)** Immunoblotting of p-STAT3 (Tyr705), STAT3, p-STAT1 (Tyr701), STAT1, p-GSK3β (Ser9) and GSK3β in treated macrophages of each groups. Data expressed as mean ± S.E.M. Each diamond represents one biological replicate. *p < 0.05; **p < 0.01; ***p < 0.001; ****p < 0.0001 (One-way ANOVA with Tukey’s Test).

High concentrations of FN showed no significant inhibitory effect on macrophage proliferation (**Figure 7A**). We induced M1-type polarization of macrophages *in vitro* by combination of LPS (50 ng/ml) and IFN-γ (40 ng/ml), followed by the treatment with FN (80 or 160μM) for 24 h. Dual immunofluorescence and flow cytometry were employed to assess macrophage polarization status. The decreased iNOS fluorescence intensity (**Figure 7B**) and reduced proportion of CD86^+^ cells (**Figure 7C**) collectively indicate FN-mediated inhibition of macrophage M1 polarization. Unexpectedly, while FN decreased mRNA levels of certain M1 polarization markers (*iNOS*, *IL-1β* and *IL-6*), it up-regulated others (*TNF-α*, *CXCL10* and *Dectin-1*) (**Figure 7D**) and concurrently reduced CD206 fluorescence intensity (**Figure 7B**). The expression of *VEGF-C* decreased as expected, but polarization markers for M2 (*CD206*, *Arg-1* and *IL-10*) remained unchanged (**Figure 7D**).

Furthermore, we investigated the STAT1 and STAT3 signaling pathways, which are strongly associated to macrophage polarization. The combined use of LPS and IFN-γ significantly promoted STAT1 and STAT3 expression and induced their phosphorylation (**Figure 7E**). We found that FN treatment reduced the protein levels of total STAT1 and dose-dependently inhibited the phosphorylation of STAT3 (**Figure 7E**). In line with previous studies (13), we validated that FN induced inhibitory phosphorylation of GSK3β (Ser9), which contributes to inhibition of M1 macrophage polarization.

### FN inhibits hLEC proliferation and modulates the paracrine effects of M1 macrophages on hLECs

**Figure 8:**
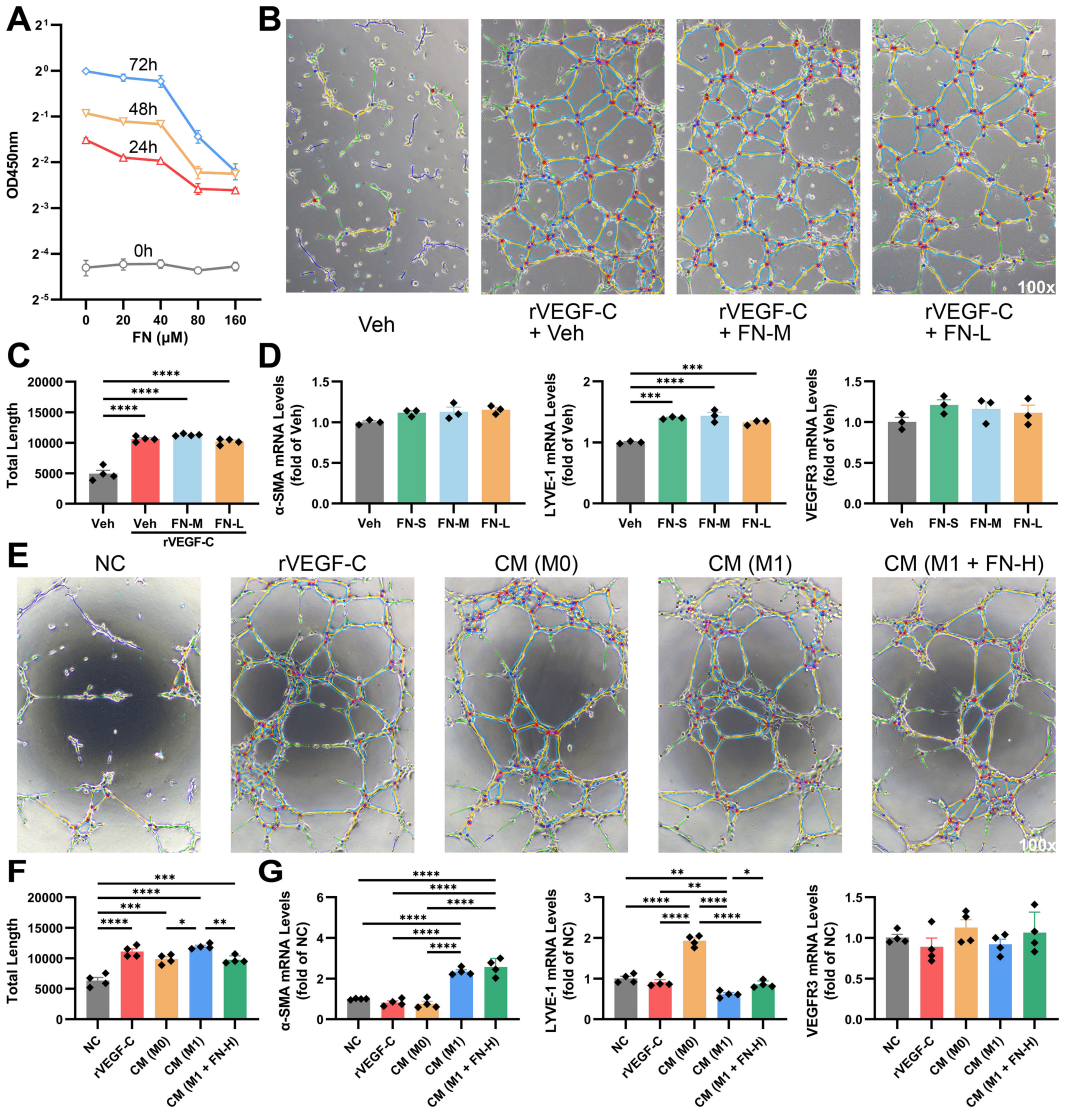
FN modulates lymphangiogenesis through direct action and macrophage polarization-mediated indirect effects. hLECs were exposed to FN at different concentrations to assess direct effects, and were incubated with conditioned mediums from FN-treated macrophages to evaluate indirect effects. FN-S, 20 μM; FN-M, 40 μM; FN-L, 80 μM; FN-H, 160 μM; CM, 1:1 mixture of conditioned medium and negative control medium. **(A)** CCK-8 assay of hLECs treated with FN (0-160 μM, 0–72 h). **(B, C)** Representative light microscopy images of tube formation assay in FN-treated hLECs and quantitative analysis of the total length of tubes. **(D)** The mRNA expression of *α-SMA*, *LYVE-1* and *VEGFR3* in hLECs treated with varying concentrations of FN. **(E, F)** Representative light microscopy images of tube formation assay in conditioned medium-treated hLECs and quantitative analysis of the total length of tubes. **(G)** The mRNA expression of *α-SMA*, L*YVE-1* and *VEGFR3* in hLECs treated with different conditioned mediums. Data expressed as mean ± S.E.M. Each diamond represents one biological replicate. *p < 0.05; **p < 0.01; ***p < 0.001; ****p < 0.0001 (One-way ANOVA with Tukey’s Test).

At relatively high concentrations, FN significantly inhibits hLEC proliferation (IC50 < 80μM), which contrasts sharply with its lack of effect on HSCs and macrophages (**Figure 8A**). The tube formation assay of hLEC, the vital tool for studying lymphangiogenesis *in vitro*, was conducted to further investigate the direct effects of FN on hLECs. When rVEGF-C was added as a positive stimulant, the quantitative analysis of the total length of tubes demonstrated that FN did not inhibit lymphatic tube formation (**Figures 8B, C**). Additionally, FN did not significantly affect α-SMA or VEGFR3 mRNA levels and even increased LYVE-1 mRNA expression in hLECs (**Figure 8D**). These findings suggest that FN does not restrict hLEC proliferation by interfering with the VEGF-C/VEGFR3 pathway.

Conditioned mediums produced by differently-treated macrophages, were applied in the tube formation assay of hLECs to evaluate the paracrine effects of macrophages on hLECs. M1 macrophages performed best in lymphangiogenesis, which was impeded by FN treatment (**Figures 8E, F**). Interestingly, we found these conditioned mediums having disparate impacts on the expression of *α-SMA* and *LYVE-1*. Conditioned medium from M1-type macrophages notably promoted *α-SMA* expression and decreased *LYVE-1* expression, while those from M0-type macrophages dramatically upregulated *LYVE-1* expression (**Figure 8G**). The impairment of *LYVE-1* expression from M1 macrophage was reversed by FN, but no significant change was observed in *α-SMA* expression. These suggest that polarization status will significantly influence macrophage-hLEC interactions.

## Discussion

Our study demonstrates that FN treatment effectively ameliorates cirrhosis-induced portal hypertension in rats, as evidenced by reduced portal pressure without affecting mean arterial pressure. As a multi-target natural compound, the therapeutic mechanisms of FN were systematically investigated in the liver, with particular focus on hepatic fibrosis and macrophage M1 polarization. The results revealed that FN significantly inhibits the activation of intrahepatic SMAD3, STAT1, and STAT3 signaling pathways. These observations were further validated *in vitro*, where FN potently suppressed both TGF-β1-induced HSC activation and LPS/IFN-γ-mediated macrophage M1 polarization. This multi-target action underscores the translational potential of FN for repurposing in portal hypertension management.

To establish experimental parameters, we first determined optimal FN dosing for both *in vivo* and *in vitro* studies through systematic literature evaluation. Although FN is clinically approved as an adjunct therapy for menopausal symptoms, its application in hepatic disorders remains unexplored. Preclinical toxicity data indicate a mouse acute dose of 300 mg/kg, with LD50 and NOAEL values of 103.6 mg/kg and 50 mg/kg, respectively (14). Previous rat studies employed varying FN doses (10–100 mg/kg/day) (15–17). Given the marked pathology in BDL-induced PHT, we selected an intermediate dose (50 mg/kg/day), which yielded significant therapeutic benefits in portal hypertensive rats.

Initial findings confirmed the therapeutic potential of FN in PHT rat models, demonstrating significant improvements in both portal pressure and hepatic function, thereby providing the rationale for further investigation. Building upon prior evidence that FN mitigates renal fibrosis through SMAD3/ATF3/SLC7A11 pathway inhibition (18), we extended these observations to hepatic fibrosis using comprehensive analyses. Histopathological evaluation (H&E, Sirius red, and Masson’s trichrome staining) combined with molecular assessment of α-SMA and COL1A1 expression at transcriptional and translational levels unequivocally established the potent anti-fibrotic activity of FN in liver tissue. HSC activation, mediated through TGF-β/SMAD, Notch, Wnt/β-catenin, and Hedgehog signaling pathways (19), represents a pivotal mechanism in fibrogenesis. This study employed TGF-β1 to activate HSCs, followed by FN treatment. The experimental results revealed that TGF-β1 stimulation significantly up-regulated the protein levels of downstream signaling molecules SMAD2 and SMAD3 with promoted phosphorylation, and increased the expression of activation markers, including *α-SMA*, *COL1A1*, *TGF-β1*, and *PDGFRβ*. FN dose-dependently reduced the expression of two core activation markers, α-SMA and COL1A1, and significantly decreased the protein expression of p-SMAD3 and SMAD3, but not SMAD2. The similar phenomenon was observed *in vivo*.

Notably, while FN downregulates TGF-β1 expression in carotid arteries (20), our BDL model and HSC experiments detected no significant changes in TGF-β1 protein isoforms (precursor, monomeric, or dimeric forms) following FN administration. This tissue-specific differential response likely reflects distinct cellular sources and activation mechanisms of TGF-β1 in hepatic versus extrahepatic systems. Importantly, accumulating evidence (21, 22) consistently supports the anti-fibrotic efficacy of FN in liver disease, reinforcing its therapeutic potential.

Expanding our investigation beyond HSCs, we explored the immunomodulatory effects of FN on hepatic macrophages. While FN has demonstrated macrophage regulatory properties in colitis, coronary artery disease, and renal injury models (23–25), its hepatic effects remain poorly characterized. Our immunohistochemical and dual immunofluorescence analyses revealed that FN reduces hepatic macrophage infiltration and modulates M1/M2 polarization in PHT rats, consistent with its known immunomodulatory activity. However, its effects on inflammatory cytokines yielded complex and sometimes contradictory findings. Although FN potently suppressed *IL-1β* expression in M1-polarized macrophages, it failed to significantly alter hepatic IL-1β, its receptor, or antagonist levels *in vivo*. FN showed no inhibitory effect on *TNF-α* expression in either activated HSCs or M1-polarized macrophages *in vitro*, yet reduced hepatic *TNF-α* levels. Intriguingly, FN differentially regulated M1 markers, downregulating *iNOS*, *IL-1β* and *IL-6* while upregulating *TNF-α*, *CXCL10* and *Dectin-1*. Among anti-inflammatory cytokines, FN selectively increased *IL-10* but decreased *IL-4* expression. These pleiotropic effects likely reflect the ability of FN to coordinately regulate multiple signaling pathways and elicit cell-type-specific responses across intrahepatic cell populations.

Further characterization confirmed the anti-inflammatory properties of FN in PHT. Immunofluorescence co-staining and flow cytometry demonstrated FN-mediated reduction in iNOS fluorescence intensity and CD86^+^ cell populations. The STAT family plays a pivotal role in macrophage polarization. STAT1 is essential for IFN-γ-induced M1 macrophage polarization (26), while STAT3 influences macrophage polarization in both directions (27). As expected, FN reduced the LPS/IFN-γ-induced upregulation of both total STAT1 and STAT3 proteins, along with p-STAT3. Previous studies indicated that FN also inhibited M1 macrophage polarization by modulating the GSK3β signaling pathway (13). We experimentally validated this conclusion and demonstrated that FN significantly enhances the inhibitory phosphorylation ratio of GSK3β. Additionally, FN demonstrates inhibitory effects on IL-6 secretion by activated HSCs. Collectively, despite generating some paradoxical effects due to its pleiotropic nature, FN ultimately exerts anti-inflammatory actions in PHT and suppresses M1 macrophage polarization.

The lymphatic system functions as both an immune cell trafficking network and a dynamic structure influenced by inflammatory processes. Previous researches suggest that M1 macrophages dominate lymphangiogenesis by secreting VEGF-C and transdifferentiating into lymphatic endothelial cells (LECs) (28), whereas M2 macrophages are associated with lymphangiogenesis in tumor development (29). The VEGF-C/VEGFR3 axis represents a critical regulator of lymphangiogenesis in both embryonic and mature tissues (30). The effects of FN on hepatic macrophages has sparked our investigative interest in intrahepatic lymphangiogenesis. Notably, evaluating hepatic lymphangiogenesis presents unique methodological challenges, as conventional lymphatic markers (LYVE-1, podoplanin, Prox1 and VEGFR3) exhibit overlapping expression with other hepatic cell types (31). Consequently, bulk quantification of these markers at either mRNA or protein levels lacks specificity for true lymphangiogenic activity. To overcome this limitation, we employed dual immunofluorescence staining targeting LYVE-1^+^/α-SMA^-^ luminal structures in portal tracts for precise quantification. Unsurprisingly, our PHT model exhibited robust lymphangiogenesis, which FN treatment significantly attenuated. Subsequent analysis revealed that FN effectively reduced the driving force of lymphangiogenesis, as evidenced by decreased VEGF-C and VEGF-D levels in both hepatic tissue and circulation.

Our findings raise two pivotal translational questions: (1) What is the mechanistic basis for FN-mediated lymphangiogenesis suppression, and (2) Does lymphatic inhibition compromise the therapeutic efficacy of FN in PHT? Given the established anti-angiogenic effects of FN via FGF2 targeting (11), we first examined its direct action on LECs. FN demonstrated potent, concentration-dependent anti-proliferative effects on LECs, while showing minimal impact on HSCs or macrophages, suggesting endothelial-specific activity. M1-polarized macrophages exhibited robust VEGF-C mRNA upregulation, which FN treatment significantly attenuated. This was functionally validated by tube formation assays, where conditioned medium from FN-treated M1 macrophages showed reduced pro-lymphangiogenic activity, compared to untreated controls. While FN clearly inhibits lymphangiogenesis, the functional implications require careful evaluation. The role of lymphangiogenesis in hepatic pathology remains controversial, with emerging evidence suggesting that LECs may contribute to periportal fibrogenesis through upregulated expression of extracellular matrix genes (*COL1A1* and *COL5A1*) in fibrotic and cholestatic models (32). Importantly, lymphangiogenesis suppression may simply reflect FN-mediated amelioration of the primary hepatic pathology, the driver of pathological lymphatic proliferation. Interestingly, co-culture with conditioned medium from M1 macrophages significantly impaired the expression of the *LYVE-1*, a marker of LEC and upregulated the expression of *α-SMA*, a marker of myofibroblast, in LECs. This suggests that macrophages interact with LECs beyond merely providing VEGF-C/D. Notably, FN not only preserved VEGFR3 expression but also upregulated LYVE-1 expression in LECs. In tube formation assays, FN did not impair the pro-lymphangiogenic function of rVEGF-C, indicating preserved VEGF-C/VEGFR3 signaling. Collectively, our data suggest that FN-mediated lymphangiogenesis inhibition likely does not compromise its therapeutic efficacy in PHT. Rather, combinatorial approaches pairing FN with pro-lymphangiogenic interventions may offer superior therapeutic outcomes.

Several limitations should be acknowledged in this study. First, the lack of primary cell validation may affect the physiological relevance of our *in vitro* observations. Second, while the BDL model primarily represents cholestatic liver injury, the therapeutic effects of FN requires systematic evaluation in other etiologies of hepatic fibrosis. Third, although we have identified the phenotypic effects of FN on multiple cell types, the underlying molecular mechanisms warrant further investigation. Finally, while we validated macrophage-LEC interactions *in vitro*, their *in vivo* relevance, particularly regarding M1-mediated LEC dysfunction under pathological conditions, remains unclear.

In conclusion, our study establishes the therapeutic potential of FN for cirrhotic PHT through multi-modal mechanisms. Using both rodent models and cell line systems, we demonstrate that FN exerts: (1) potent anti-fibrotic effects, (2) macrophage polarization modulation, and (3) lymphangiogenesis inhibition. These findings position FN as a promising multi-target therapeutic candidate for portal hypertension management.

## Data Availability

The original contributions presented in the study are included in the article/**Supplementary Material**. Further inquiries can be directed to the corresponding authors.

## Glossary

ALT: alanine transaminase
AST: aspartate Aminotransferase
BDL: bile duct ligation
CM: conditioned media
CMC-Na: carboxymethylcellulose sodium salt
ECM: endothelial cell medium
ELISA: enzyme-linked immunosorbent assay
FN: formononetin
hLEC: human lymphatic endothelial cells
HSC: hepatic stellate cell
IHC: immunohistochemistry
IF: immunofluorescence
LEC: lymphatic endothelial cells
LSEC: liver sinusoidal endothelial cell
LVA: lymphatic vessel area
LVD: lymphatic vessel density
MAP: mean arterial pressure
PHT: portal hypertension
PP: portal pressure
PV: portal vein
PVDF: polyvinylidene fluoride
RT-qPCR: real-time quantitative polymerase chain reaction
SD: Sprague Dawley
SDS-PAGE: sodium dodecyl sulfate - polyacrylamide gel electrophoresis
TBIL: total bilirubin
