# Supplementary material for: Therapeutic potential of formononetin in cirrhotic portal hypertension: modulating hepatic fibrosis, macrophage polarization, and lymphangiogenesis
Source: Front Immunol. 2025 Aug 7;16:1571007. doi: 10.3389/fimmu.2025.1571007 (PMC12367483; doi:10.3389/fimmu.2025.1571007)

**Supplementary Table. S1**

The primers used for reverse transcription and quantitative PCR reactions.

| Gene | Forward primer sequence (5'-3') | Reverse primer sequence (5'-3') |  |
| --- | --- | --- | --- |
| Human *GAPDH* | TCGGAGTCAACGGATTTGGT | TGAAGGGGTCATTGATGGCA |  |
| Human *α-SMA* | CTATGAGGGCTATGCCTTGCC | GCTCAGCAGTAGTAACGAAGGA |  |
| Human *LYVE-1* | AATTTCACAGAAGCTAAGGAGGC | TCAAGGCTGTTTCAACTTGGTC |  |
| Human *VEGFR3* | TGCACGAGGTACATGCCAAC | GCTGCTCAAAGTCTCTCACGAA |  |
| Mouse *GAPDH* | AGGTCGGTGTGAACGGATTTG | TGTAGACCATGTAGTTGAGGTCA |  |
| Mouse *iNOS* | CAGCACAGGAAATGTTTCAGC | TAGCCAGCGTACCGGATGA |  |
| Mouse *IL-1β* | AGTTGACGGACCCCAAAAG | AGCTGGATGCTCTCATCAGG |  |
| Mouse *IL-6* | ACAACCACGGCCTTCCCTAC | TCTCATTTCCACGATTTCCCAG |  |
| Mouse *TNF-α* | CTCTTCTGTCTACTGAACTTCGG | AAGATGATCTGAGTGTGAGGGT |  |
| Mouse *CXCL10* | GCTGCCGTCATTTTCTGC | TCTCACTGGCCCGTCATC |  |
| Mouse *Dectin-1* | CTTCACCTTGGAGGCCCATT | TCGCCAAAATGCTAGGGCA |  |
| Mouse *CD206* | GAGGGAAGCGAGAGATTATGGA | GCCTGATGCCAGGTTAAAGCA |  |
| Mouse *Arg-1* | CCACAGTCTGGCAGTTGGAAG | GGTTGTCAGGGGAGTGTTGATG |  |
| Mouse *IL-10* | GCTGCGGACTGCCTTCA | TGCATTAAGGAGTCGGTTAGCA |  |
| Mouse *VEGF-C* | TCTGTGTCCAGCGTAGATGAG | GTCCCCTGTCCTGGTATTGAG |  |
| Rat *GAPDH* | TTGTGCAGTGCCAGCCTC | GGTAACCAGGCGTCCGATAC |  |
| Rat *α-SMA* | AGTCGCCATCAGGAACCTCG | GCCATTGTCACACACCAGAG |  |
| Rat *COL1A1* | TGGTACATCAGCCCAAACCC | CGCTTCCATACTCGAACTGG |  |
| Rat *TGF-β1* | CTTGCCCTCTACAACCAACA | ACTTGCGACCCACGTAGTAGA |  |
| Rat *PDGFRβ* | TTCCAGAGGTGATGCCAGCTT | AGGGGGCGTGATGACTAGG |  |
| Rat *IL-1β* | CTATGGCAACTGTCCCTGAA | GGCTTGGAAGCAATCCTTAATC |  |
| Rat *IL-4* | CGTGATGTACCTCCGTGCTT | GTGAGTTCAGACCGCTGACA |  |
| Rat *IL-6* | ATAGTCCTTCCTACCCCAATTTCC | GATGAATTGGATGGTCTTGGTCC |  |
| Rat *IL-10* | CGCTGTCATCGATTTCTCCC | GACACCTTTGTCTTGGAGCTTAT |  |
| Rat *IL-1R1* | GGTTTAGCTCCGGGTTTAGC | CTTTCATATTCTCCTGGGCGT |  |
| Rat *IL-1Ra* | TCCTTCTCATCCTTCTGTTTCGT | ATTCTGAAGGCTTGCATCTTGC |  |
| Rat *TNF-α* | GCCTCAGCCTCTTCTCATTC | GGGAACTTCTCCTCCTTGTTG |  |
| Rat *VEGF-C* | TGCCGGTGCATGTCTAAA | CTGCCTGACACTGTGGTAAT |  |
| Rat *VEGF-D* | TGGGACAGAAGACCACTCTTA | TCCAGGACATGGTGCTTTAC |  |

**Supplementary Fig. S1**

Gating strategy with quantitative outcomes

(a) Debris exclusion by FSC-A/SSC-A.

(b) Single-cell selection (FSC-H vs FSC-A).

(c) Live cell identification(FVS780^-^)

(d) Target population gating (CD86^+^/CD206^+^)


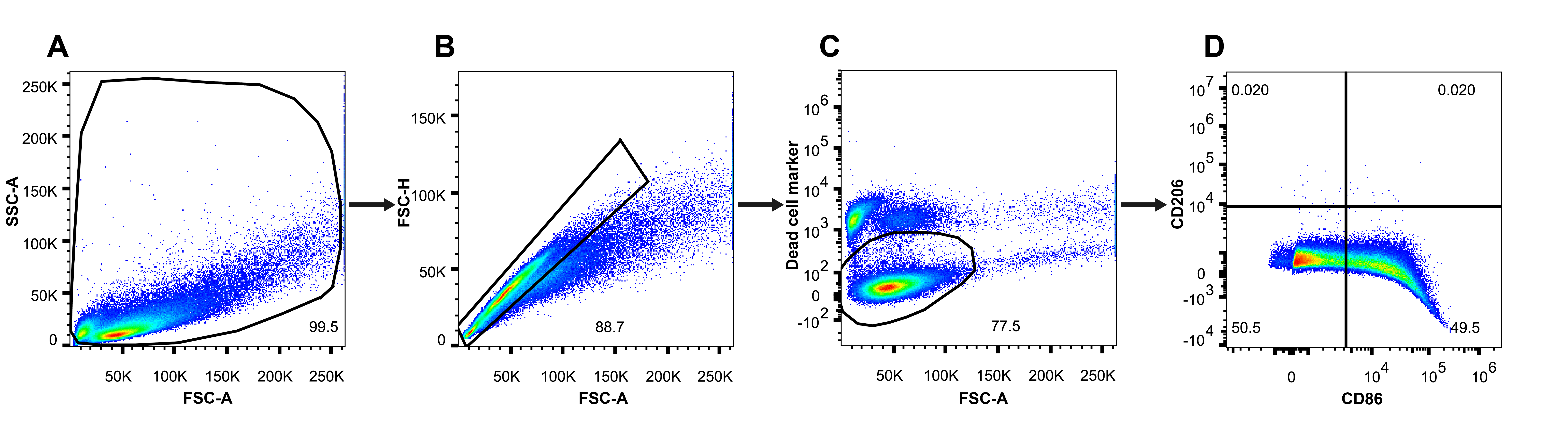


**Supplementary Fig. S2**

The mRNA expression of *IL-1R* and *IL-1Ra* in liver tissues of each groups.


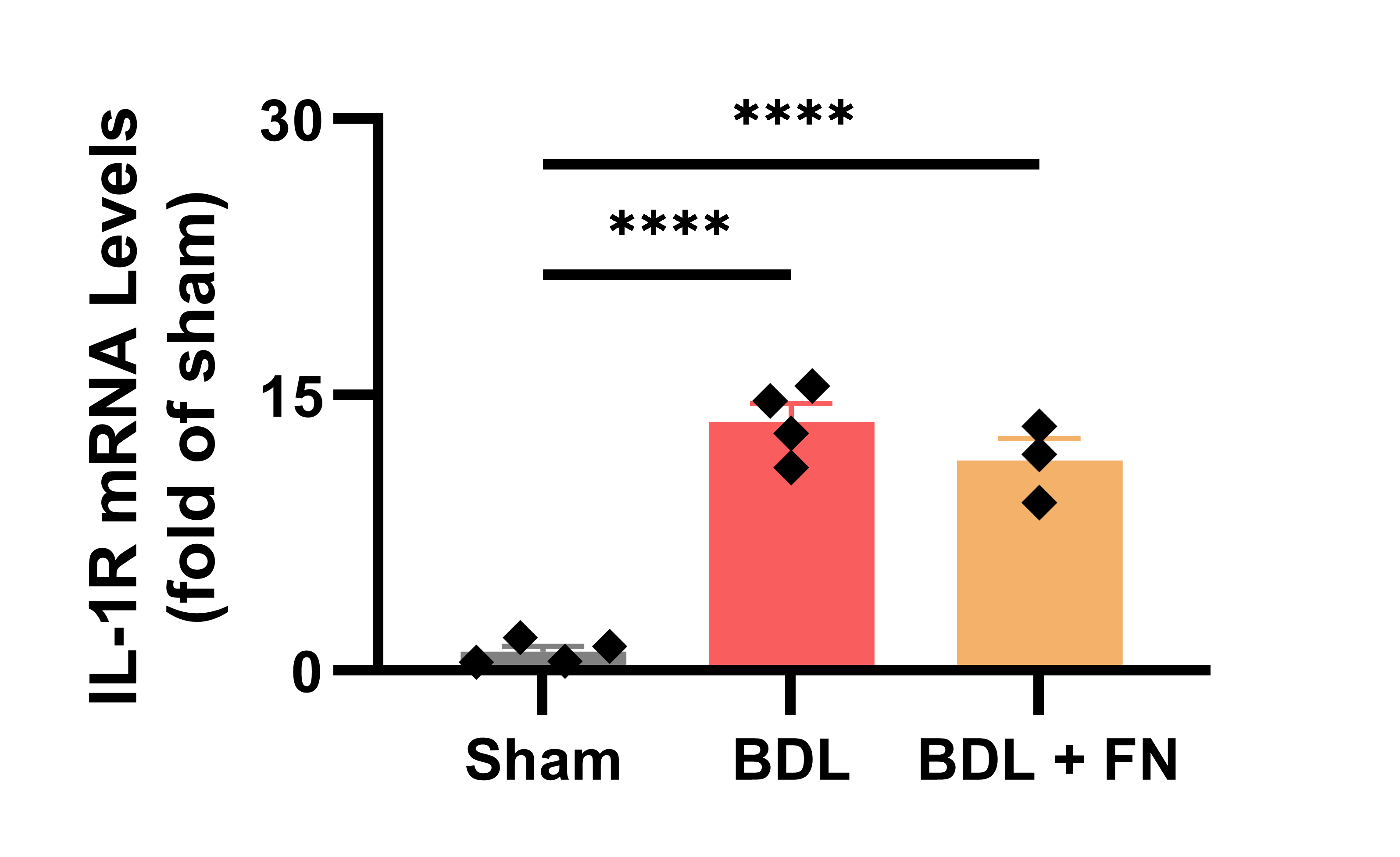

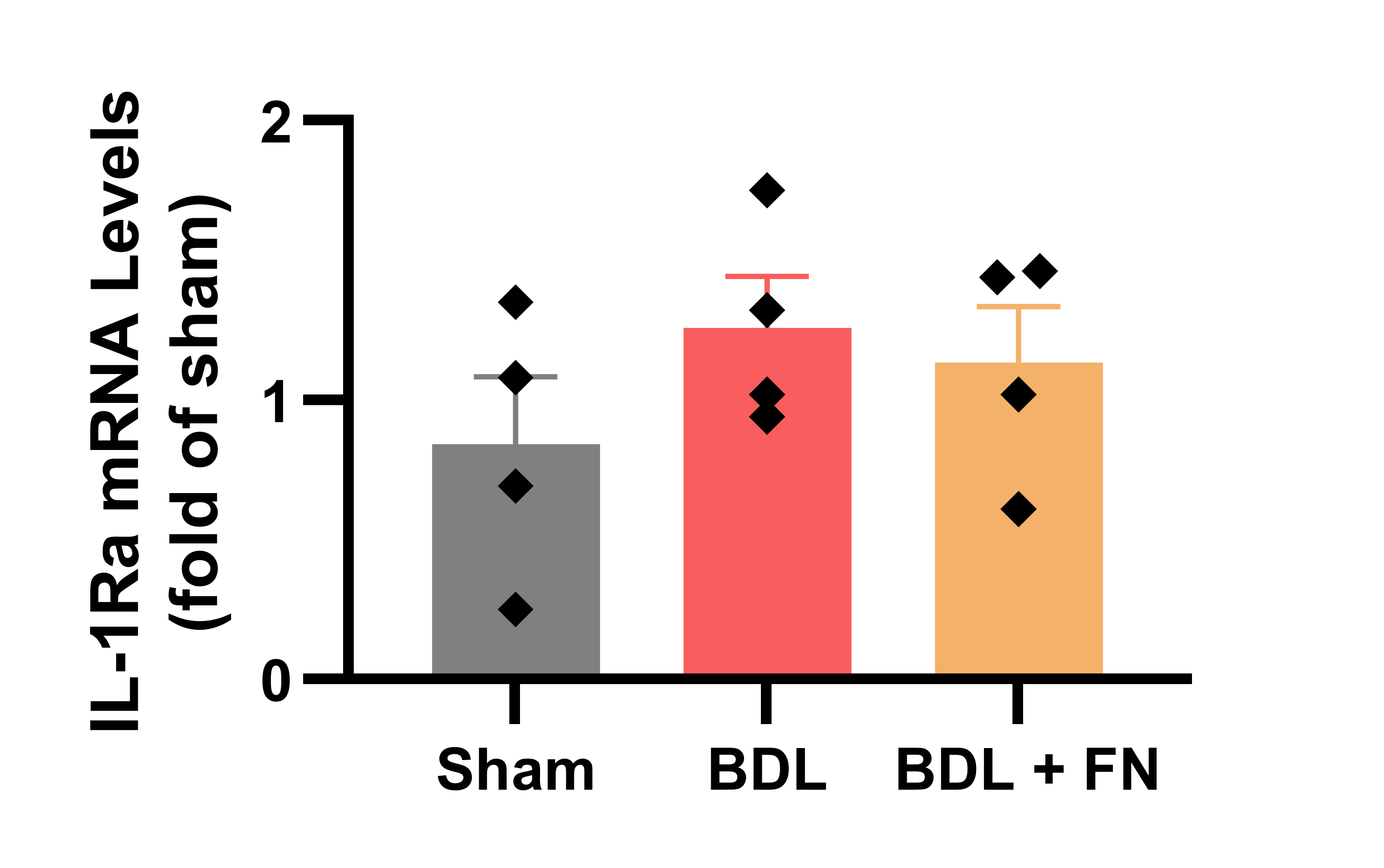

Supplement: Supplementary file 1 [file Supplementaryfile1.doc]
